# Microscopic description for the emergence of collective dissipation in extended quantum systems

**DOI:** 10.1038/srep42050

**Published:** 2017-02-08

**Authors:** Fernando Galve, Antonio Mandarino, Matteo G. A. Paris, Claudia Benedetti, Roberta Zambrini

**Affiliations:** 1 Instituto de Física Interdisciplinar y Sistemas Complejos IFISC (CSIC-UIB), Campus Universitat Illes Balears, E-07122 Palma de Mallorca, Spain; 2 Quantum Technology Lab, Dipartimento di Fisica, Università degli Studi di Milano, I-20133 Milan, Italy; 3 Center for Theoretical Physics, Polish Academy of Sciences, Aleja Lotników 32/44, 02-668 Warszawa, Poland

## Abstract

Practical implementations of quantum technology are limited by unavoidable effects of decoherence and dissipation. With achieved experimental control for individual atoms and photons, more complex platforms composed by several units can be assembled enabling distinctive forms of dissipation and decoherence, in independent heat baths or collectively into a common bath, with dramatic consequences for the preservation of quantum coherence. The cross-over between these two regimes has been widely attributed in the literature to the system units being farther apart than the bath’s correlation length. Starting from a microscopic model of a structured environment (a crystal) sensed by two bosonic probes, here we show the failure of such conceptual relation, and identify the exact physical mechanism underlying this cross-over, displaying a sharp contrast between dephasing and dissipative baths. Depending on the frequency of the system and, crucially, on its orientation with respect to the crystal axes, collective dissipation becomes possible for very large distances between probes, opening new avenues to deal with decoherence in phononic baths.

Models for quantum dissipation address the interaction of a quantum system with bosonic, fermionic or other kinds of environments, where the relevant information about the microscopic structure of the environment is encoded in its spectral density^1^,^2^,^3^,^4^. On the other hand, further information is required to properly describe spatially extended multipartite systems: an often used generalization is the independent dissipation of the system’s components into separate baths (SB), leading to complete erasure of quantum correlations^1^,^2^. Also, collective or spatially symmetric decoherence into a common bath (CB)^5^,^6^,^7^,^8^,^9^,^10^,^11^,^12^,^13^,^14^,^15^,^16^,^17^,^18^,^19^,^20^,^21^,^22^,^23^,^24^ has been proposed as an alternative scenario in the limit of small system size (or components separation) in comparison with environment correlation length or with radiating atoms’ transition wave-length^2^,^5^,^6^. A CB opens up outstanding possibilities like superradiance^2^,^10^, superdecoherence^5^, and decoherence free/noiseless subspaces^11^,^12^, allowing the preservation and also creation of entanglement^13^,^14^,^15^,^16^,^17^,^18^, the emergence of collective synchronization^19^, with potential applications in quantum computation^7^,^8^,^9^,^20^,^21^,^22^,^23^ and metrology^24^.

Besides artificial methods to engineer collective dissipation mechanisms^25^,^26^, the cross-over between CB to SB can naturally arise in structured environments. The still open and fundamental question is: *how small needs to be a multipartite system to dissipate collectively*? The CB/SB cross-over when increasing the size of spatially extended systems has been phenomenologically modeled in the last decade yielding a smooth change and, generally, assuming isotropic dispersion relations of bosonic environments^27^,^28^,^29^,^30^ (like it happens for electromagnetic radiation in free-space^2^,^6^). Assuming a distance dependent transition from collective to independent dissipation, important predictions have been reported in the context of quantum error correction^31^, in the dynamics of photosynthetic complexes^32^,^33^,^34^ and in quantum metrology^35^. Even if a microscopic derivation of the CB/SB cross-over is *still missing* in spatially structured environments, it is usually argued that a common environmental medium with significant spatial correlations up to distances *ξ*_*c*_ will produce both damping for each system unit and a *cross-damping* among them: a collective dissipation is therefore generally associated to systems smaller than the correlation length *ξ*_*c*_, while units far away will be damped independently in SB. Here we are going to show the failure of this prediction for a large class of energy-matter exchange dissipation models, particularizing to a specific microscopic model to clarify and illustrate several details: a phonon bath in a crystal probed at different spatial locations. We address the cross-over from CB to SB in detail, providing a physical ground for the description of intermediate regimes, and assessing the role played by geometric factors, spatial extension of the system-probe contact and bath correlations. Our model allows to clarify several issues including: a) why when increasing the system size in 1D environments^27^,^28^,^30^,^36^ there is no asymptotic interpolation between CB and SB, but a periodic cross-over; b) why choosing an isotropic environmental dispersion relation will always lead to distance-decaying cross-damping, c) why anisotropic dispersion relations (like those in real crystals with symmetries) can lead to surprising effects like CB at large distances, also showing d) that in general the correlation length is *not* related to the CB/SB transition. We further e) give a simple intuitive picture of how a bath’s frequency cutoff appears naturally from the fact that the system’s quantum units have a finite spatial extent, and f) we show how the presence of static disorder favours SB dissipation.

For clarity we introduce next a particular model displaying all the phenomenology, and leave the discussion on the generality of these effects to the last section.

## Results

**Figure 1:**
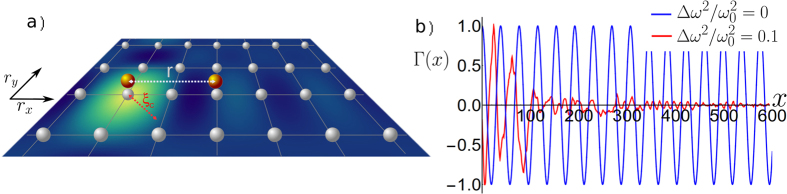
(**a**) Sketch of a 2D crystal and two locally attached probes, at distance 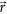. We pictorially plot the bath’s correlation (of spatial extent *ξ*_*c*_) centered in one probe (see also Fig. 5c). (**b**) Cross-talk for the 1D periodic and disordered environment as a function of the probes distance. Added random noise in the onsite potential with amplitude Δ*ω*^2^ = 0, 0.1, *ω*_0_ = 1, *g* = 3/4, probe frequency Ω resonant with *k*_Ω_ = 0.164. The normalized cross-talk in absence of noise is 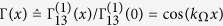 while in presence of disorder it is position dependent due to lack of translational invariance, 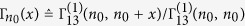. We present 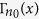 for an arbitrary *n*_0_ and a given noise realization, with *x* ∈ [0, 600] and a we have used a finite harmonic chain of 2500 oscillators.

We consider a *D*-dimensional periodic crystal, in the same spirit that led Rubin^37^ to introduce a linear harmonic chain as a microscopic model of an Ohmic bosonic bath^36^,^38^. This model allows to model spatially correlated dissipation and provides a common ground to assess the role of different crystal dimensionality D and geometries, including spatial disorder effects, either for point-like and for non-local system-bath interactions. The *D*-dimensional crystal consists on an infinite collection of harmonically coupled masses (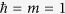) with on-site harmonic potential of frequency *ω*_0_ (see Fig. 1a for a representation for *D* = 2). We focus for the sake of simplicity in oscillations in one direction corresponding to one phonon polarization (see Methods). The dissipative system consists of two probes whose distance 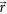 can be tuned, namely two uncoupled harmonic oscillators of frequency Ω *weakly* interacting with the crystal. We start considering point-like contacts at two different spatial locations 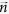 and 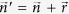.

The master equation^2^ for the reduced density matrix of the two probes may be obtained within the Born-Markov approximation and assuming the environment in a Gibbs state at temperature *T*


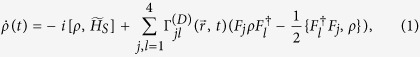


where 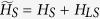, with *H*_*S*_ and *H*_*LS*_ the system Hamiltonian and bath’s Lamb-shift (see equation (9) in Methods). The 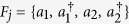 are the annihilation (creation) operators of each probe and 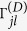 are the corresponding damping coefficients (the superscript refers to the dimensionality of the crystal), depending only on the distance 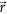 owing to environment translational invariance. Self-damping of each oscillator (*j* = *l*) and cross terms (|* j* − *l*| = 2) characterize the dissipation with


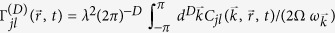


and non-vanishing terms


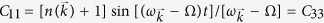



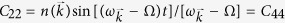



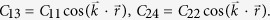


For a bath at zero temperature, the only nonzero coefficients are the the self-damping 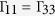 and cross term 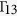.

A crucial point is that if the two probes are attached to a common environmental point (CB case), i.e. 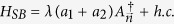, we have Γ_11_ = Γ_13_ and Γ_22_ = Γ_24_^30^, whereas for probes attached to two independent environments (SB case) we would have Γ_13_ = Γ_24_ = 0, i.e. no cross terms. The cross-over between CB and SB regimes depending on the probes distance can now be derived from this microscopic model without further assumptions. For long times, when the weak dissipation becomes important, only a family of resonant momentum crystal phonons are relevant, such that 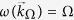. This condition identifies the manifold of phonons mediating an eventual cross-talk between the oscillators. The dependence on probes distance at *T* = 0 and long times is then


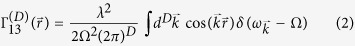


Note that in the weak damping regime we are considering here, the dissipation rate *λ*^2^ is much smaller than the frequencies of the problem, which guarantees that at times where the quantum units start ‘feeling’ dissipation, the sinc function is well approximated by a delta.

### The exceptional 1D case and disorder effects

Notice that an immediate consequence, previously observed in refs 27,28,30, but scarcely commented upon, is that for 1D homogeneous environments, irrespective of the dispersion relation, we have 
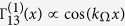
, since the frequency resonance constraint exhausts all freedom in choosing the crystal momenta in eq. (2). This means that two probes will experience collective dissipation not only when attached to the same point of the environment but also when at the anti-nodes of the resonant mode^36^. In this case the relative position or the center of mass of the pair is shielded from decoherence, allowing to preserve entanglement among the probes at large distances. Indeed the surprising results is the lack of asymptotic cross-damping decay above any distance, being the cross-over between SB and CB periodically predicted. Further, if the relative size of cross-damping and self-damping are considered, this result is unchanged when increasing the temperature of the thermal bath (this is due to 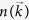 factoring out of the integrals because it depends only on the frequency).

The generalization to higher dimensional environment leads to a richer scenario, but before proceeding it is interesting to assess the fragility of this phenomenon in experiments considering the effect of *static disorder*. The cross-talk can be understood as the sum of overlaps of resonant crystal normal modes at the probes positions. The expression (2) obtained when plane waves are the normal modes, can be in general expressed as


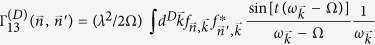


where 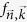 is the spatial profile of eigenmode 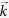, and now the cross-talk is position dependent (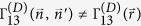). The presence of disorder, here modeled by inhomogeneity in the local crystal potentials, breaks translational invariance and leads to localized waves. As a consequence the cross-talk, periodic in the homogeneous case, now decays with the distance at an average rate depending on the degree of disorder, as shown in Fig. 1b. This localization effect^39^ hinders the periodic cross-over between CB and SB leading to a spatial decay: beyond some distance, two independent probes will dissipate into SB.

### Isotropic vs. anisotropic cases

When moving to *D* > 1 a common assumption in several phenomenological approaches, either based on spin-boson^6^,^23^,^27^,^28^,^30^,^35^ or boson-boson models^29^, is the isotropy of the dispersion relation of the environment, i.e. its dependence only on the modulus 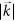. This is the case for electromagnetic environment^2^. The isotropy of the environment dispersion enables some analytical insight and leads to a *spatially decaying* cross-talk in the master equation. For *T* = 0 and long times the cross-talk dependence on the environment dimension is


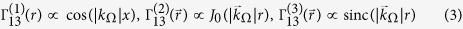



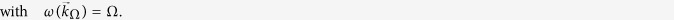


**Figure 2:**
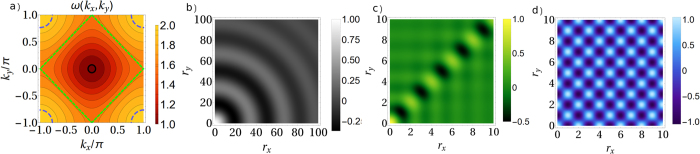
(**a**) 2D dispersion relation in color code with *ω*_0_ = 1 and *g* = 3/16, so that 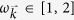. Iso-frequency surfaces are shown for the limiting cases discussed in the text: black Ω = 1.01 corresponding to the isotropic case, green) 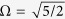 and blue Ω = 1.95. Normalized cross-damping term 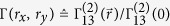 for (**b**) the isotropic case (low momenta), for (**c**) directional non-decay (medium momenta) and (**d**) non-decay (high momenta) (see text for details). We plot only one spatial quadrant because of the symmetry of the setting.

On the other hand the dispersion in spatially structured media are typically not isotropic. In the case of a cubic homogeneous crystal, for instance,


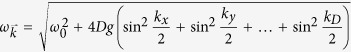


where we recognize the effect of the spatial symmetries (we discuss later the triangular case). Still the dispersion is approximately isotropic for small momenta (Fig. 2a black circle) 
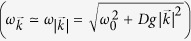
, and the angular integration yields a function decaying with the radial distance between probes (Fig. 2b). Independently on the crystal direction probed by the system components, collective dissipation is lost above some distance where the crystal will effectively acts as two SB.

Departure from isotropic dispersion relations has deep consequences. Although in general there will be a spatial (non-monotonic) decay of 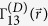, different scenarios may arise like those of Fig. 2c and d. In general the anisotropy of the dispersion will translate into a sensitivity of the probes dissipation to the crystal geometry. In Fig. 2c we observe for a particular resonance value Ω an interference effect resulting in decay of 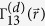 along all directions *except* for the lattice diagonals *y* = ±*x* where it does not decay. Indeed 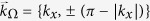 yields 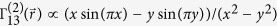, not decaying on the crystal diagonals. Strong anisotropy is also displayed in Fig. 2d, for *k*_*x*_ = ±*π, k*_*y*_ = ±*π* and leading to a periodic cross-term 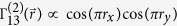. Then no asymptotic decay of the cross-damping with distance occurs and these high frequency probes are able to ‘resolve’ the spatial structure of the crystal.

Similar results are found in 3D: resonant momenta for a given Ω will lie in a surface, and cross-talk will depend on their interference. For isotropic (low momenta) case we have the form 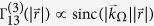, while for high momentum we have a similar ‘egg-crate’ in 3D 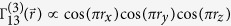. Also the 2D peculiar case of Fig. 2c has an analog here with non-decaying crossover along diagonal directions.

### Other crystal symmetries

**Figure 3:**
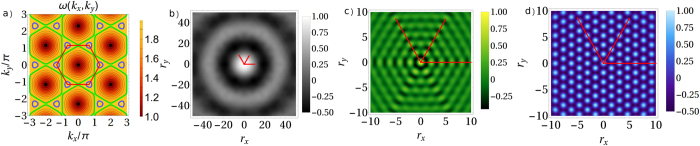
(**a**) 2D dispersion relation in colour code with *ω*_0_ = 1 and *g* = 0.165, so that 
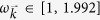
. Iso-frequency surfaces are shown for the limiting cases equivalent to those of the cubic crystal of the main text: black Ω = 1.01 corresponding to the isotropic case, green Ω = 1.905 directional non-decay, and blue) Ω = 1.99 non-decay. We have also plotted in red the fundamental (Wigner-Seitz) cell, to which momentum integrals are restricted. Normalized cross-damping term 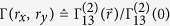 for (**b**) the isotropic case (low momenta), for (**c**) directional non-decay (medium momenta) and (**d**) non-decay (high momenta), where we have added in red the crystal symmetry directions to show that the cross-damping term conserves the symmetry of the problem.

Our predictions are robust also in different geometries as for example in the *triangular lattice* (instead of cubic). In this case diagonalization of *H*_*B*_ would be done through plane waves along momentum directions corresponding to the correct Bravais lattice. Since the direct lattice has proper vectors (in 2D now for simplicity) 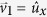 and 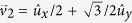, its Bravais lattice has vectors 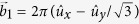 and 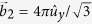. The momentum expansion should be done in this directions and the dispersion relation results


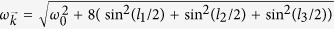


with *l*_1_ = *k*_*x*_, 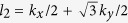 and 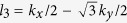. The behaviour of dissipation displays (see Fig. 3) the same regimes of decaying cross-talk for low momenta, and non-decaying cross-talk for higher momenta along symmetry-favoured directions.

### Short time behaviour

**Figure 4:**
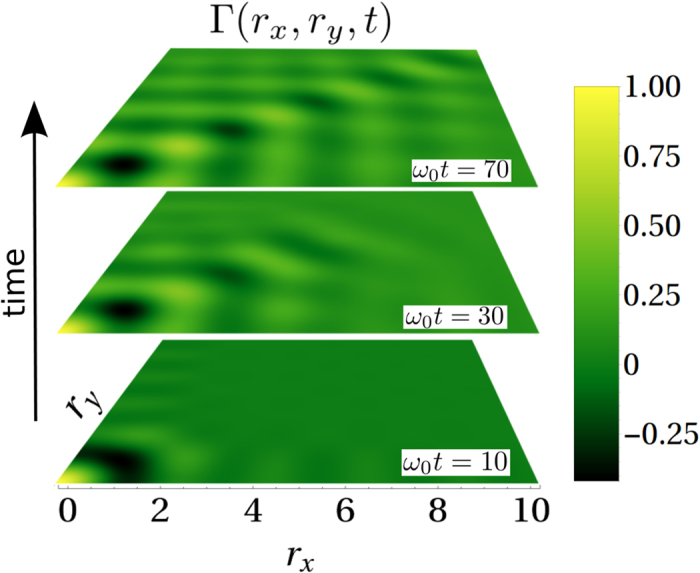
Short-time behaviour of the 2D crystal cross-talk, for the case (**c**) of Fig. 2 in main text for times (**a**) *ω*_0_*t* = 10,30,70. The long time limit corresponds to (Fig. 2c).

So far we have discussed the long time limit, relevant for the weak coupling regime, whereas at *short times* there is a transient in which the signal travels from one probe to the other at the crystal’s fastest group velocity and no cross-damping exist. This is seen in the cross-talk, which expands its spatial structure at that velocity (see Fig. 4 and Supp. Inf.), reaching its final (momentum dependent) form (displayed for *t* → ∞ in Figs 1, 2 and 3).

### Finite temperature

The damping coefficients typically associated with cooling (Γ_11_ and Γ_13_) and heating (Γ_22_ and Γ_24_), have a temperature dependence which is encoded in the factors 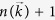 and 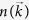, respectively, which for bosonic modes are given by the relation 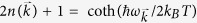. For weak damping, the resonant filtering of frequencies (which transforms the sinc function into a delta in frequencies), transforms this 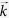-dependent factor in the integral into a purely numeric factor 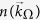, weighting the population of bath modes at the given frequency Ω. So in comparison with *T* = 0 where Γ_22_ = Γ_24_ = 0, now also these heating terms are present, with a factor 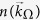, whereas the cooling terms (Γ_11_ and Γ_13_), go with a factor 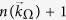. The spatial behaviour is thus unchanged.

If damping is not so weak, at early times there is still the effect of the 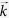-dependent sinc function and the number occupations of each mode 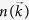, which add different weightings to each mode inside the integral. These weights give more importance to low-frequency modes in the bath, although we have checked that the net effect on the damping rates is not too pronounced.

### Extended spatial coupling

Considering probes with a *finite spatial extension* and hence coupled to a finite-sized region of the crystal, instead of single atoms, elucidates the meaning and presence of frequency (momentum) cut-off *ω*_*c*_ in the description of open systems. Even if a crystal presents a natural maximum frequency determined by its periodicity, in open systems the cut-off is often not a property of the environment^29^, depending instead on the probe system. Let us consider probes with extended interaction 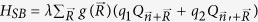 with 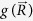 a function decaying for 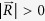 up to each probe size. The new cross-term integrand 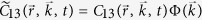 is modified by a contact form factor 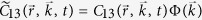 and the long times, *T* = 0, new expression reads


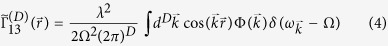


where 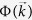 limits the maximum effective wavenumbers. For a system-probe coupling 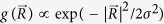, the factor 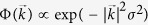 leads to filtered integrals, stemming from the fact that a probe of spatial size *σ* detects an average effect on that area and will be unable to feel the influence of phonons of shorter wavelengths (higher momentum than 1/*σ*). In practice, in order to reach the situation in Fig. 2d each probe needs to have a spatial extent smaller than the crystal spacing, so that it senses the highest available phonon momenta (*σ* → 0, so 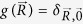).

### Correlation length in the crystal

**Figure 5:**
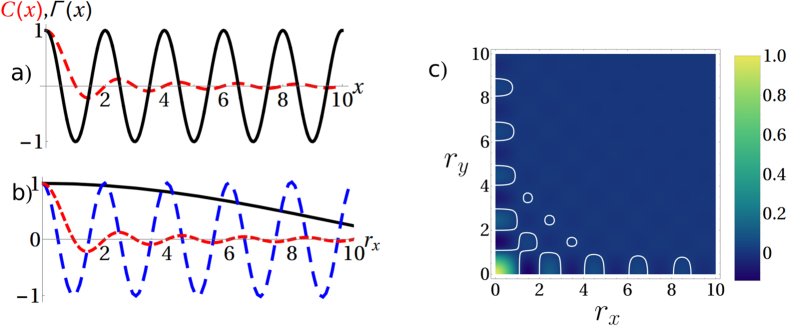
We compare here the normalized correlation function *C(r*_*x*_, *r*_*y*_)/*C*(0, 0) with the cross-damping in several cases where their decays do not match at all: (**a**) Crystal correlation function *C(x*) in 1D in red, vs. the cross-damping term in black of probes with frequency Ω = 2*ω*_0_. We have chosen *ω*_0_ = 1 and 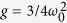, so that again 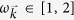. Lower Ω would simply resonate with a lower momentum and we would see a cosine with longer periodicity. (**b**) Correlation function for the 2D-crystal in red, compared with the cross-damping along *r*_*x*_ (with *r*_*y*_ = 0) for the isotropic case (black) and high momentum case (blue), as previously shown in Fig. 1b and d, respectively, with the same parameters as Fig. 1. (**c**) *C(r*_*x*_, *r*_*y*_) in colour code, and we have highlighted the particular value *C(r*_*x*_, *r*_*y*_) = 0.01 in white to guide the eye. This shape does not change significantly for higher temperatures (see Supp. Inf.). Further, the short range is not peculiar of this crystal symmetry: a similar behaviour can be observed for the triangular crystal (see Supp. Inf.).

Does the transition from CB to SB we have seen up to now have to do with the correlation length of the environment? The quick answer is no, as can be seen in Fig. 5a and b. The cross- and self-damping terms in the dissipation equation (1) come from bath operator spatial correlation functions 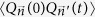 at two times. This time dependence is the one that, for long times, selects a unique wave vector 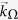 due to resonance with Ω (through the factor 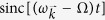) and therefore follows from a reduced manifold *D*-1 of momenta. On the other hand, the correlation in the crystal at two different points comes from functions at equal time 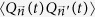 and follows from all phonons momenta. In other words cross-damping is caused by *resonant* phonons, while generic correlations in the crystal are caused by interference of *all* phonons thus decaying with distance even in 1D (Fig. 5a). Usually, as in our case, the bath is in a stationary (thermal) state, and thus the correlation function is time-independent 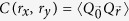, with 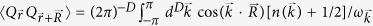. The 2D case (Fig. 5b and c) clearly displays spatial correlations decay at distances of the order of the crystal lattice 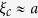 (notice that all spatial coordinates are scaled with *a* in the rest of the manuscript), being stronger along crystal directions, while the cross-talk decays on a scale given by the resonant normal mode wave-length 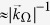 (isotropic case) or does not decay at all (anisotropic case).

## Discussion

### Generality

Our conclusions can be generalized to other system-bath models, e.g. where the environment is a non-interacting field which exchanges excitations with the system probes: i.e a collection of free particles with a given dispersion relation 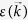 whose eigenfunctions have a spatial profile 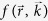, so that the bath Hamiltonian is 
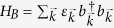
 (or 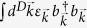 for continuous spectra); the exchange interaction between bath and system probes being 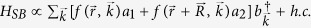, so probe 1 is located at 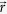 and probe 2 at 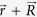. In such case and assuming secular and Born-Markov regime, the cross-talk is given by


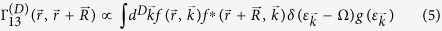


where the function 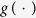 is related to how the probes couple to each mode. The free particles could be Bogoliubov bosons on top of a condensate in an optical lattice, electrons in the bulk, phonons in a crystal with disorder (as in the main text) or any other free particles which, because of the locality and weakness of the probe-bath coupling lead to such master equation with this cross-term. The delta function is a consequence of the fact that the system-bath is energy exchanging, and thus that we have *dissipation*. Note also that we have assumed that different bath modes are uncorrelated and stationary, as usual in e.g. a thermal state.

The bath free field can be expressed in terms of the single-particle operators, leading to a correlation function


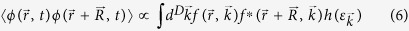


(notice that there are two generic functions *h* and *g* which are model-dependent). For non-interacting fields it is thus clear that CB/SB cross-over distance is unrelated to the correlation length in the medium, simply because the former is propagated by resonant free particles, while the latter is propagated by all particles. Other models with more complicated interactions than just particle exchange, or even interacting models for the bath, might yield different behaviours and are subject of future interest.

We comment now on how the phenomenology studied for the D-dimensional crystal translates into this generic class of models: a) in the 1D case the cross-damping will be of the form 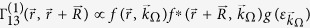, meaning that the overlap of mode functions (of eigenmode 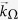) between the two probe positions will dictate the decay from CB to SB, i.e. the spatial shape of 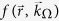, be it localized or periodic, will lead to decay or non-decay respectively; b) the very peculiar behaviour observed in Fig. 2c,d requires very well-matched interference of plane waves (thus a translational invariant medium) and thus is not to be expected in general; c) the short/long time argument is based on the nature of the *sinc* function and thus independent on the details of the model, hence any possible long-range cross-damping will take a time to build up, related with the fastest excitations in the environment; finally, d) also irrespective of the details of the bath model, the presence of a probe with finite spatial extension will blur any short-range (high momentum) details, leading to a high-momentum cutoff in the integrals defining the coefficients of the master equation.

### Dephasing

A further interesting point is to consider the comparison with the case of a *dephasing model*. In that situation the cross-talk will *not* have any resonance constraint imposed, and thus the two integrals eqs (5) and (6) will be similar except for the functions 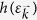 and 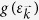, leading to similar behaviours. Some typical bath’s spectral densities (encoding function *g*(·) and the density of states of the bath) *ω*^*D*^ exp(−*ω/ω*_*c*_) favour small momenta in the cross-talk for 1D, while for 3D they favour frequencies/momenta near the cut-off frequency *ω*_*c*_^5^,^27^. Thus for pure dephasing the CB to SB transition length will be similar to the correlation length of the environment.

### Experimental implementations

One possible way to experimentally implement the 2D crystal is via trapped ions with a tight axial confinement so that they effectively lie on a plane and form a triangular-symmetric Coulomb crystal, such as in ref.^40^. The major problem in that setting is that axial motion is coupled to radial degrees of motion, but this can be overcome if the axial frequency is sufficiently higher than the radial counterpart. The probe ions would need to be sitting in the same plane thus distorting the modes of the Coulomb crystal. Therefore the modelling would be slightly more complicated. although the basic physics would be the same. Addressability of the probe ions, e.g. by fluorescence 41, would be a central requirement.

Another possible way of investigation is the intentional deposition of atoms adsorbed in metallic surfaces. This has always been considered as a drawback and a source of anomalous heating in ion trap electrodes^3^,^42^, but could suit our purposes. Adsorbed atoms bound to a metallic surface can have oscillation frequencies in the THz regime, very close to Debye frequencies of metals (gold for example has a Debye frequency of around 3.6 THz). In this way, by placing intentionally adsorbed atoms at different distances would allow us to check our results. Different masses of these atoms would scan the different frequencies as compared to the maximum phonon frequency of the metallic substrate. For this to be possible we should deal with fluorescent adatoms which can be addressed and localized by lasers. Investigation of cross-damping could be done by exciting the motion of one atom and evaluating the effect on the other. A coupling of the fluorescent transition to the motional degree of freedom would probably be needed, though.

### Outlook

An immediate consequence of this work is that initial correlations between two dissipating units will be highly sensitive to details of the underlying medium, such as crystal symmetries. This suggests a possible avenue to use multi-party quantum systems to test/probe media with unknown properties. One could further envision the use of a lattice of coupled probes to obtain information of an unknown surface through the decay of spatial modes of the probe-lattice. In this direction, recent work^43^ has shown that a single trapped ion can be confined near a metallic surface to extract electric-field noise characteristics through its heating rate. Also, in view of recent proposals to use surface acoustic waves as a quantum bus between many different types of quantum systems^44^, the phenomenon of preferential directions seen in Figs 2 and 4 could be potentially used to build substrates with a patterned surface whose symmetry allows for distant units to communicate along diagonal/triangular directions with a decay only given by static imperfection (disorder) of the material. All these avenues are left for future investigation.

## Conclusion

Do the separate units of a spatially extended system suffer dissipation and decoherence from common or separate baths? We tackled this fundamental issue introducing a microscopic environment model where spatial distances and correlations appear naturally. Beside the ineffectiveness of environment spatial correlations to determine this transition, we have shown the importance of dimensionality, symmetries and probes extensions. The prediction of collective dissipation between distant probes in a 1D homogeneous environment when placed at a distance multiple of 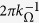 opens up interesting possibilities in surface phononic cavities^44^ and phonon wave-guides^45^. Similar predictions can hold for planar or bulk platforms environments, for probes at relative position now determined both by their oscillations frequency and the crystal symmetries. Indeed when *D* > 1, the dispersion is isotropic for 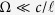, with *c* the effective propagation velocity in the medium and 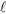 either wavelength of the crystal periodicity or the mean distance between disorder patches in an otherwise homogeneous medium^3^. The anisotropy opens a communication channel (resulting from the interference of a manifold of resonant phonons) between the probes, even at large distance (see Figs 2c,d and 3c,d) while the effect is degraded in presence of disorder. On the other hand, independent dissipation (SB) will occur for rather distant and ‘slowly oscillating’ probes, when the effective dispersion is isotropic (see Figs 2b and 3b) as in the largely studied case of electromagnetic fields in homogeneous media.

Collective and local dissipation of multipartite systems in crystal environments can be extended to frontline platforms that can serve as substrates in quantum technologies, such as metamaterials with gapped spectra or displaying topological modes^46^,^47^, and in polaritons configurations^48^, optomechanical arrays^49^,^50^ or cold atoms in different phases^51^. Furthermore, even if disorder in 1D environments has been shown to hinder collective dissipation, there are several open questions in larger dimensions and in presence of phenomena such as Anderson localization^52^.

## Methods

### Master equation in periodic and disordered environment

We consider a *D*-dimensional harmonic crystal with nearest neighbour interactions: 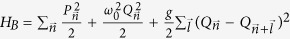 where 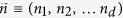 is the site index where each mass lies, and 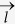 are unit lattice vectors, being for a cubic structure 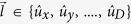 in each of the *D* spatial directions. The probes are first considered as point-like coupled to the bath at points 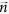 and 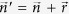, so the system-bath interaction is 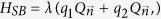. The overall Hamiltonian is given by *H* = *H*_*B*_ + *H*_*S*_ + *H*_*SB*_ where the extended system consists of the two identical uncoupled 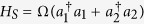 harmonic probes. Notice that we introduce only one degree of freedom for each site, which corresponds also to a model of a scalar field with spatial discrete structure. If we set *ω*_0_ = 0, in 3D it also can be associated to studying cross-talk mediated by phonons of only one polarization in a realistic crystal, as for example gold^53^, with a linear anisotropic dispersion that saturates for high momenta. Since dissipation into the crystal can always be decomposed into three polarizations, we can choose to match the probe-to-probe direction, thus separating the problem into the three sets of polarizations, each having an anisotropic dispersion relation, as here considered.

The master equation of the system (two probes) density matrix up to the second order in the coupling strength, is obtained in the Born-Markov approximation^2^ and given by


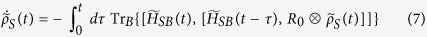


in the interaction picture 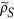, where 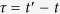 and 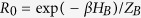 the invariant thermal state of the (crystal) environment. In the crystalline case, the bath Hamiltonian is diagonalized by plane waves, and the system-bath Hamiltonian is then


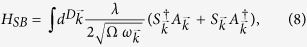


with 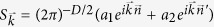. In the case of a crystal with disorder, translational invariance is broken and the bath is not any more diagonalized by plane waves, but by the general transformation 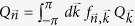 and the system operators read 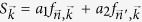.

After some standard algebraic operations, and going back to Schrödinger picture, the master equation reduces to


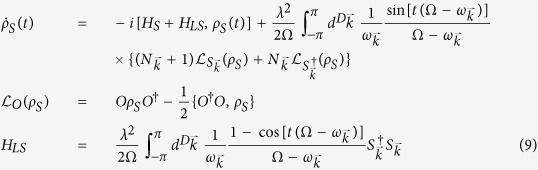


with 
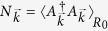
, and substituting the corresponding operators 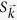 in the equations. In terms of 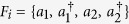, the dissipative part reads 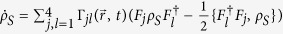, with


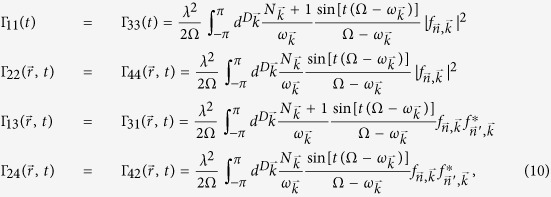


always understanding that 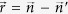. Correspondingly :


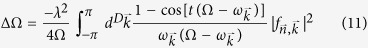



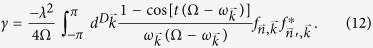


In the crystalline case (no static disorder), we have 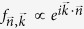, so 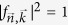 and 
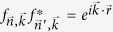
 (which leads, throught the symmetry of 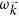 to 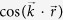 in the main text).

## Additional Information

**How to cite this article:** Galve, F. *et al*. Microscopic description for the emergence of collective dissipation in extended quantum systems. *Sci. Rep.* **7**, 42050; doi: 10.1038/srep42050 (2017).

**Publisher's note:** Springer Nature remains neutral with regard to jurisdictional claims in published maps and institutional affiliations.

## Supplementary Material

Supplementary Information
