# Supplementary material for: Microscopic description for the emergence of collective dissipation in extended quantum systems
Source: Sci Rep. 2017 Feb 8;7:42050. doi: 10.1038/srep42050 (PMC5296766; doi:10.1038/srep42050)
Supplement: Supplementary Information [file srep42050-s1.pdf]

Microscopic description for the emergence of collective dissipation in extended quantum systems

Fernando Galve[1*] and Antonio Mandarino[2], Matteo G. A. Paris[2], Claudia Benedetti[2], Roberta Zambrini[1]
[1]*Instituto de Física Interdisciplinar y Sistemas Complejos IFISC (CSIC-UIB), Campus Universitat Illes Balears, E-07122 Palma de Mallorca, Spain and*
[2]*Quantum Technology Lab, Dipartimento di Fisica, Università degli Studi di Milano, I-20133 Milan, Italy*

Short time behaviour

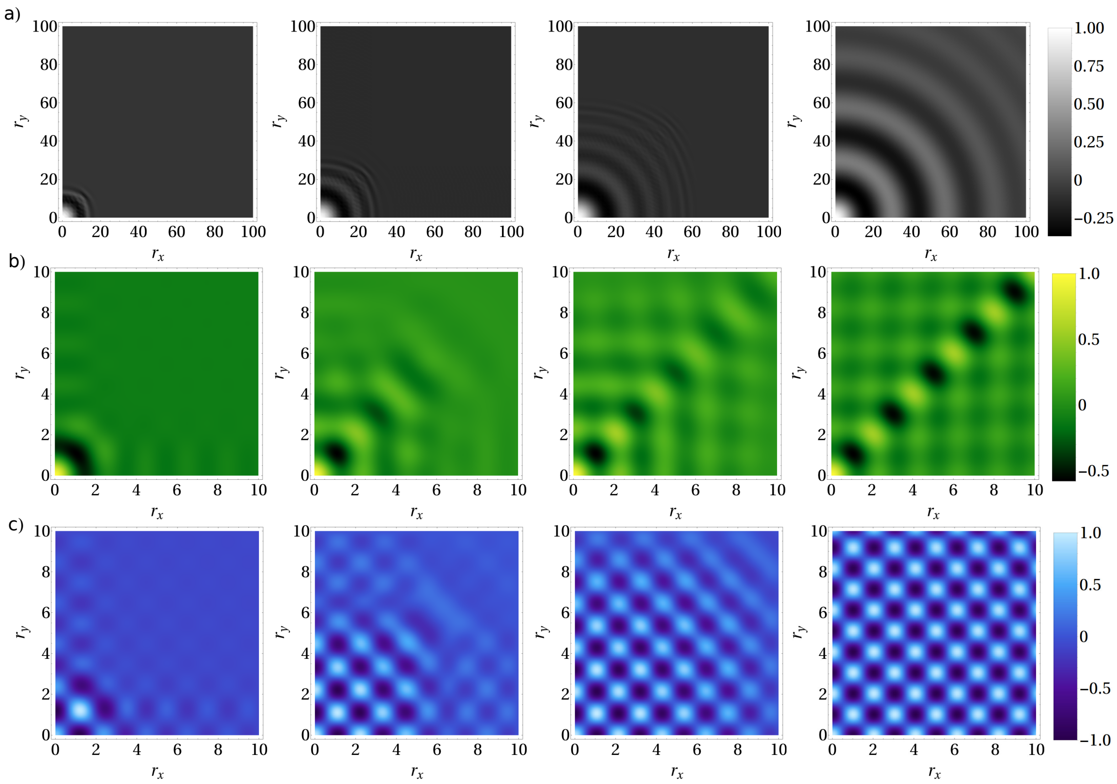

FIG. 1: Short-time behaviour of the 2D crystal cross-talk, for the limiting cases of figure 2 in main text: a) isotropic, b) directional non-decay, c) non-decay. From left to right, we show times a) $\omega_0 t = 50, 100, 200, 1000$ and b,c) $\omega_0 t = 10, 30, 70, 10000$. For b) and c) it was necessary to plot longer times in order to see better the resulting cross-talk obtained in main text's figure 2 for the long time limit.

*fernando@ifisc.uib-csic.es

Correlation function for finite temperature

The environment's correlation function at finite temperature is

$$\left\langle \phi(\vec{r})\phi(\vec{r}+\vec{R})\right\rangle \propto \int_0^\pi d^D\vec{k}\left[2n(\vec{k})+1\right]\cos(\vec{k}\cdot\vec{R})$$
$$=\int_0^\pi d^D\vec{k}\coth\left[\frac{\hbar\omega_{\vec{k}}}{2k_BT}\right]\cos(\vec{k}\cdot\vec{R})$$

and therefore the temperature dependence contributes to its spatial shape. This is in contrast with the long-times behaviour of the cross-talk at finite temperature

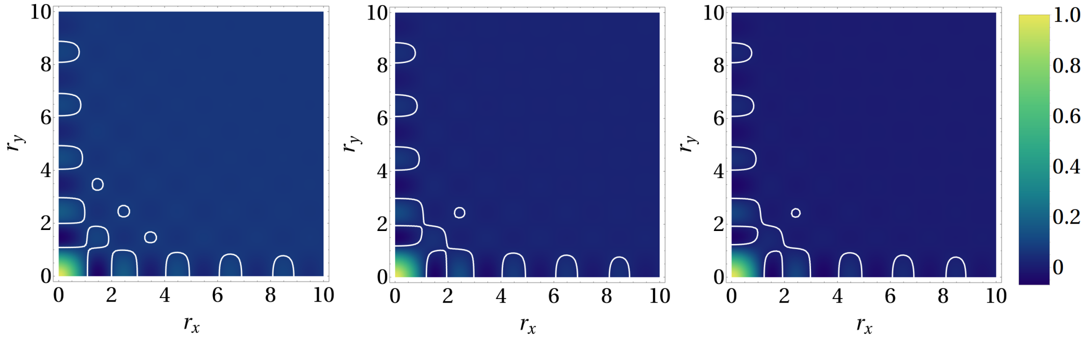

FIG. 2: Normalized correlation function at finite temperature $C(\vec{r},T)$ for the 2D cubic crystal in colour code. From left to right, $T/\omega_0 = 0, 1, 100$. We stress the prominently weak influence of temperature on most features, specially its spatial distribution. We note that for the case where the substrate is Gold, with Debye ω_D frequency around THz regime, in real units we would have $k_BT/\hbar\omega_D = 0, 1, 100$ corresponding to $T = 0, 10, 1000$ degrees Kelvin.

$$\Gamma_{13}^{(D)}(\vec{r}) \propto \int_{-\pi}^{\pi} d^D\vec{k}\delta(\omega_{\vec{k}}-\Omega)\left[n(\vec{k})+1\right]\cos(\vec{k}\cdot\vec{R})$$
$$=\int_{-\pi}^{\pi} d^D\vec{k}\delta(\omega_{\vec{k}}-\Omega)\frac{1}{2}\left[\coth\left(\frac{\hbar\omega_{\vec{k}}}{2k_BT}\right)+1\right]\cos(\vec{k}\cdot\vec{R})$$

where the cotangent factors out of the integral. Thus we have a common prefactor $(1/2)\left[\coth(\hbar\Omega/2k_BT)+1\right]$ and an integral in momenta which does not depend on temperature, meaning that the spatial shape is independent of temperature. It must be stressed though, as seen in Fig. 2, that the correlation function is not too different for different temperature scales: its spatial decay basically coincides with the crystal constant.

Correlation function with triangular symmetry

The correlation function respects the symmetric directions of the crystal but also decays fast (on the order of the crystal spacing) as in the case of cubic symmetry.

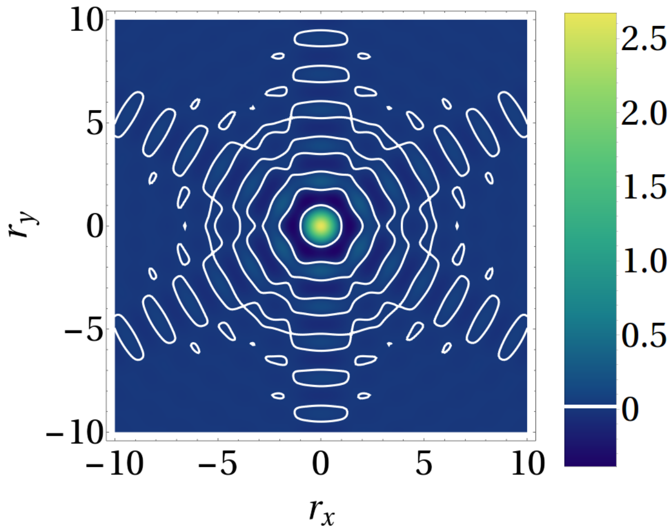

FIG. 3: Correlation function $C(r_x, r_y)$ for the 2D triangular crystal in colour code. We have highlighted the particular value $C(r_x, r_y) = 0.01$ in white to guide the eye.
